# Predicting Beef Fatty Acid Composition from Diet and Plasma Profiles Using Multivariate Models

**DOI:** 10.3390/ani15202969

**Published:** 2025-10-14

**Authors:** Marco Acciaro, Leonardo Sulas, Gianfranca Carta, Sebastiano Banni, Elisabetta Murru, Claudia Manca, Corrado Dimauro, Myriam Fiori, Andrea Cabiddu, Giovanni Antonio Re, Maria Giovanna Molinu, Giovanna Piluzza, Valeria Giovanetti

**Affiliations:** 1 AGRIS Sardegna, S.S. Sassari-Fertilia 291, km 18.6, 07100 Sassari, Italy; 2 Institute for the Animal Production System in Mediterranean Environment, National Research Council, Traversa La Crucca 3, Località Baldinca, 07100 Sassari, Italy; leonardo.sulas@cnr.it (L.S.);; 3 Department of Biomedical Sciences, Section of Physiology, University of Cagliari, Cittadella Universitaria, Monserrato, 09040 Cagliari, Italybanni@unica.it (S.B.); m.elisabetta.murru@gmail.com (E.M.); claumanca@unica.it (C.M.); 4 Department of Agricultural Science, University of Sassari, Via E. de Nicola, 07100 Sassari, Italy; dimauro@uniss.it; 5 Institute of Sciences of Food Production, National Research Council, Traversa La Crucca 3, Località Baldinca, 07100 Sassari, Italy

**Keywords:** pasture, stall, phenolic compounds, fatty acids, partial least square regression, canonical correlation, plasma, meat

## Abstract

**Simple Summary:**

The nutritional quality of beef depends largely on the fats it contains, which is strongly influenced by the animals’ diet. Traditionally, evaluating these traits requires slaughtering, but this study tested a less invasive approach based on blood analysis. Young cattle were raised either on natural pastures or with hay- and concentrate-based diets. The results showed that key dietary components, especially natural antioxidants found in pasture plants and the fat fraction of the feed, play an important role in determining the meat composition. Diets richer in antioxidants were associated with higher levels of health-promoting fats, such as omega-3 fatty acids and conjugated linoleic acid, known for their benefits to human health. Blood plasma analysis proved to be a reliable predictor of these traits, allowing the meat quality to be monitored without killing the animal. This innovative strategy could help farmers improve sustainability, increase product value, and provide consumers with healthier beef.

**Abstract:**

The nutritional value of beef is highly influenced by its fatty acid composition. This study evaluated whether diet proximate analyses or plasma fatty acid profiles could predict the meat fatty acid composition in young beef cattle finished at pasture or with hay- and concentrate-based diets in stalls. Eighteen crossbred animals (Limousine × Sardo-Bruna) were analyzed for plasma and the intramuscular fat composition of *Longissimus thoracis* (LT) and *Musculus gluteus maximus* (MGM). A canonical correlation analysis revealed strong relationships between the dietary antioxidant capacity and meat lipid profiles, particularly for α-linolenic acid and conjugated linoleic acid. The redundancy index indicated that diet explained 38% of the variance in LT fatty acids and 20% in MGM. Partial least squares regression achieved a high precision and accuracy (R^2^ up to 0.94), with a low root mean square error of prediction and high predictive ability (Q^2^ > 0.85), in predicting the intramuscular fatty acid composition from plasma samples. Overall, (i) animals consuming diets with a higher antioxidant capacity and rich in n-3 precursors (ether extract) have healthier fat profiles, and (ii) plasma fatty acid profiling can be a powerful method for monitoring meat quality. This approach provides farmers with a non-invasive tool to improve meat quality management and promote healthier beef products.

## 1. Introduction

The growing consumer demand for “healthy” products may benefit traditional beef livestock farming, which relies on the suckler–cow system and rotational grazing of natural pastures in several Mediterranean areas. Typically, calves are raised with their mother at pasture until weaning, around 6–7 months of age, before being moved to fattening centers where they are finished with concentrate-, silage-, and hay-based diets [1]. Currently, the price paid for the weaned calves is considered unprofitable by farmers, while fattening centers capture most of the added value and profit [2,3]. As a result, farmers are encouraged to finish their calves on-farm using a pasture-based diet, which reduces feeding costs and potentially enhances animal welfare.

Several studies have highlighted the nutritional benefits of grassland-based beef products. Compared to most beef from intensive systems, animals raised on pasture typically have a lower fat content and a higher concentration of nutritionally valuable compounds, such as n-3 polyunsaturated fatty acids (PUFAs), conjugated linoleic acid (CLA), vitamins, and phenolic compounds [3,4,5,6]. The high biodiversity of Mediterranean pastures provides valuable sources of compounds with a nutricine value (i.e., the contribution to health promotion and disease prevention through bioactive food components) [7], such as phenolic compounds, fatty acids, and vitamins, which help protect against oxidative-stress-induced diseases in animal metabolism [8]. Phenolic compounds are influenced by plant growth and development and enhance plant metabolism [9,10]. Additionally, they play a significant role in ruminant nutrition and welfare, with possible antihelminthic, antimethanogenic, and antimicrobial effects [11,12,13].

Therefore, a proper utilization of herbage peaks from natural pastures during late spring could be crucial for the on-farm finishing of local beef cattle. Presumably, exploiting the available standing forage resources may lower the finishing feed costs and improve the beef quality.

Regarding the assessment of meat quality traits, it is important to understand the possible relationship between bioactive compounds in the animal diet and plasma fatty acid composition. Recent studies indicate significant correlations between specific plasma fatty acids and the composition of *Longissimus dorsi* muscle, adipose tissue, and liver [14]. However, information on predicting the intramuscular fat composition in meat based on plasma fatty acids and/or directly from the diet chemical composition remains limited, as predictive models are still lacking. This study may address this gap.

We hypothesized that significant advancement could be achieved by predicting the meat fatty acid composition from blood samples, as they can be obtained from cattle through simple and relatively painless procedures. Furthermore, this innovative approach could facilitate the monitoring of the meat fatty acid composition throughout the on-farm finishing phase.

In this context, multivariate statistical analysis may be highly beneficial to understanding the relationship between the quality of the diet offered to finishing animals and their intramuscular fatty acid composition. It can also help to predict the intramuscular fatty acid composition based on plasma profiles, despite the high collinearity often observed among predictors [15].

A multidisciplinary research project has been initiated in Sardinia (Italy) to evaluate the impact of beef meat derived from extensive and intensive farming systems on human metabolism. In this context, the ability to predict the meat composition, particularly the presence of molecules known to positively influence human metabolism, through blood analysis would not only provide significant scientific insights but also offer farmers a valuable tool for monitoring meat quality. This could, in turn, enhance the product value and confer a competitive advantage in the marketplace. The specific objective of this work was to predict the fatty acid composition of beef intramuscular fat using the plasma composition and proximate analyses of the herbage (pasture) and hay + concentrate (stall) diets.

## 2. Materials and Methods

The study was conducted in accordance with the European Directive 2010/63/EU and the Italian Legislative Decree 26/2014 on the protection of animals used for scientific purposes. The experimental protocol was reviewed and approved by the Institutional Animal Welfare Body (Organismo Preposto al Benessere degli Animali—OPBA) of the University of Sassari under protocol number OPBA 1169/19. The related documentation was subsequently submitted to the Italian Ministry of Health, which authorized the study under protocol number E8652.1 and authorization number 1169/2020-PR. All procedures involving animals were conducted under veterinary supervision.

### 2.1. Experimental Site and Animal Management

The field experiments were carried out in spring 2019 at three sites located in the Marghine region (Central west Sardinia, Italy), across three private beef farms that varied slightly in elevation and soil characteristics (Table 1).

After weaning, to obtain a representative overview of the finishing diets for beef cattle in the study area, 18 F1 cross-bred animals (Limousine × Sardo-Bruna) were selected from the three farms (Table 1). Of these, 8 animals were fed hay + concentrate-based diet, and 10 animals were raised on a pasture-based diet.

The study area is characterized by an extensive silvopastoral lowland system, dominated by wild pear trees and shrubs, and utilized for the traditional dairy sheep and/or suckler–cow system, with pasture serving as the primary feed source [16]. Herbage availability varies annually in terms of both total dry matter yield and its seasonal distribution, strictly depending on the amount of seasonal rainfall, with typical peaks of forage production occurring in spring. The diet administered in stall did not vary alongside the observation period, and consisted of a mixture of barley, corn, field beans, bran, and hay ad libitum.

**Table 1:** Soil, animal traits, and feed characteristics in two finishing systems: Pasture vs. Hay + Concentrate.

		Pasture Diet			Hay + Concentrate Diet
Item	Attribute	Farm1	Farm2	Farm3	Stall
Farm characteristics	Latitude/longitude	40°16′ N, 8°52′ E	40°16′ N, 8°58′ E	40°15′ N, 8°49′ E	
	Altitude (m. a.s.l.)	420	200	376	
	Soil series [17]	Rock outcrop, Lithic Xerorthents	Typic, Aquic, and Ultic Palexeralf Xerorthents Palexeralf	Rock outcrop, Lithic Xerorthents Ultic Palexeralfs	
	Sand/silt/clay (%)	64/16/14	68/12/20	46/30/24	
	pH	6.0	6.3	6.0	
Animal traits	Young bulls (n°)	-	-	3	2
	Heifers (n°)	3	4	-	6
	Live weight (LW, kg mean ± s.d.)	332 ± 28	301.5 ± 13	328 ± 46	333 ± 20
	Age (days, mean ± s.d.)	353 ± 5	347 ± 5	358 ± 6	355 ± 4
	Average stock density (kg LW ha^−1^)	332	280	218	-
Feed on offer	Natural pasture	ad libitum	ad libitum	ad libitum	-
	Hay (kg head^−1^day^−1^)	-	-	-	3
	Concentrate (kg/100 kg LW * head^−1^day^−1^)	-	-	-	1.6–1.8

* LW = Live weight.

### 2.2. Feedstuff Sampling and Chemical Analysis

In late spring, herbage mass on offer was measured by cutting 0.5 m^2^ quadrats in proportion to the grazeable area available to the animals on each farm. Ten, fifteen, and twelve 0.5 m^2^ quadrats were cut in Farm1, Farm2, and Farm3, respectively. Herbage samples were taken three days prior to the turn-off pasture for slaughter, from May 30 to 31, 2019. Each herbage sample was weighed to determine the fresh biomass on offer, then partitioned into plant botanical families (Gramineae, Leguminosae, and other dicotyledons) to assess the botanical composition. Samples were then oven-dried, as a whole, at 65 °C for approximately 72 h to determine the dry matter (DM) content. Subsequently, they were ground to pass through a 1 mm screen for the analysis of crude protein (CP), neutral detergent fiber (NDF), acid detergent fiber (ADF), acid detergent lignin (ADL), ether extract (EE), and in vitro dry matter digestibility (IVDMD) using near-infrared reflectance spectrometry (NIRS) [18]. The same analyses were performed on hay and concentrate subsamples that made up the hay + concentrate diet.

For each farm, three additional representative subsamples of fresh herbage were obtained from 0.5 m^2^ quadrats. The subsamples were immediately frozen in liquid nitrogen and stored at −20 °C, until lyophilisation with Heto Lyolab 3000 (Heto-Holten A/S, Allerød, Denmark) for 48 h at −55 °C. They were then ground into a fine powder and stored at −20 °C until analysis. Hay and concentrate samples were also ground to pass through a 1 mm screen. Sample preparation procedures were conducted according to Re et al. [19]. All samples were analyzed in triplicate.

Total phenolics (TotP), non-tannic phenolics (NTP), and tannic phenolics (TP) of extracts were determined using the Folin–Ciocalteau reagent (Merck, Milan, Italy), following previously described procedures [19]. Results were expressed as g of gallic acid equivalent (GAE) per kilogram of dry matter of plant material (g GAE kg^−1^ DM), based on a calibration curve of gallic acid (Merck, Milan, Italy). Antioxidant capacity was evaluated using ABTS ((2,2′-azinobis (3-ethylbenzothiazoline-6-sulphonic acid)) diammonium salt [19]. For each assay, 0.1 mL of appropriately diluted methanolic extracts were used. Briefly, 3.9 mL of the ABTS radical solution were mixed with the sample, and spectrophotometric readings were taken after 6 min at 734 nm. The results were expressed as Trolox equivalent antioxidant capacity (TEAC), in mmol Trolox equivalents per 100 g of dry weight of leaves (mmol TEAC 100 g^−1^ DW), based on a calibration curve of Trolox (6-hydroxy-2,5,7,8-tetramethylchroman-2-carboxylic acid) (Merck, Milan, Italy).

### 2.3. Blood Sampling

Upon reaching the full growth as scheduled under the experimental conditions, and immediately before slaughtering (around 08:00 h in the morning), a blood sample was withdrawn from the caudal vein of each animal using 10 mL vacuum collection tubes containing EDTA K3 (Vacutainer Systems Europe; Becton Dickinson, Meylan Cedex, France). Immediately after collection, blood samples were cooled to 4 °C and centrifuged at 1500× *g* for 15 min. Plasma was then removed and stored at −80 °C until analysis.

### 2.4. Slaughter and Meat Sampling

At the designated sacrifice age (475 ± 45 days old, mean ± sd), grazing animals were removed from the pasture and, along with stalled animals, they were gathered and transported to an authorized commercial abattoir. Slaughtering was carried out within 1 h of arrival to minimize pre-slaughter stress. Animals were stunned using a captive bolt pistol and dressed according to standard commercial practices.

At 24 h post-mortem, samples of *Longissimus thoracis* (LT), taken between the 6th and 8th ribs, and *Musculus gluteus maximus* (MGM) from each left half-carcass were collected, homogenized, vacuum-packaged, and frozen at −80 °C until chemical analysis.

### 2.5. Plasma and Meat Fatty Acid Analysis

Plasma samples were added to a chloroform–methanol (Merck, Milan, Italy) 2:1 solution (vol/vol) containing 0.2 µg/mL of α-tocopherol for lipid extraction [20]. To obtain free fatty acids for GC and HPLC analysis, aliquots of chloroform were dried and mildly saponified as described by Banni et al. [21]. Fatty acids (FAs) were measured as fatty acid methyl esters (FAMEs), by a GC (Agilent, Model 6890, Palo Alto, CA, USA) equipped with a flame ionization detector (FID); the split ratio was set at 20:1; the injection port temperature was 270 °C; an autosampler from Agilent (Model 7673, Palo Alto, CA, USA) and a 100 m HP-88 fused capillary column (Agilent, Palo Alto, CA, USA) were used [22]. Unsaturated conjugated diene FAs (CLA and metabolites) were detected at 234 nm [23] and further identification of unsaturated FAs was performed at 200 nm [24]. Spectra (195–315 nm) of the eluate were obtained every 1.28 s and electronically stored. Separation of FAs was performed using an Agilent 1100 HPLC system (Palo Alto, CA, USA) equipped with a diode array detector. A C-18 Inertsil 5 ODS-2 Chrompack column (Chrompack International BV, Middleburg, The Netherlands) with 5 μm particle size and 150 × 4.6 mm was used with a mobile phase of CH_3_CN/H_2_O/CH_3_COOH (Merck, Milan, Italy) (70/30/0.12, *v*/*v*/*v*) at a flow rate of 1.5 mL/min. Data were acquired by the Agilent OpenLAB CDS software system, version 2.4”, Agilent Technologies.

The extraction of fatty acids from intramuscular fat of slaughtered animals was conducted similarly to that of plasma (P-FA).

### 2.6. Statistical Analysis

Difference between the fatty acids composition of plasma, LT, and MGM was analyzed by the following ANOVA model (lm procedure of R):

Yij = Sj + εij

Yij represents the fatty acid variable for the j-th observation in the i-th source (plasma, LT, or MGM), Sj is the fixed effect of the source (plasma, LT, or MGM), which is considered as a categorical factor in the analysis, and εij is the residual error term associated with the j-th observation within the i-th source, assumed to be normally distributed with mean 0 and constant variance. Treatment differences with a *p*-value ≤ 0.05 were considered as significantly different, unless indicated otherwise.

All variables were standardized prior to analysis, i.e., centered to zero mean and scaled to unit variance, so that predictors expressed in different units contributed equally to the CCA and PLSR models.

Canonical correlation analysis (CCA) [25] was employed to explore the relationships between offered diet quality data and meat FA composition of LT (LT-FA) and MGM (MGM-FA) muscles, respectively. The CCA is a multivariate extension of correlation analysis designed to investigate the complex interactions between two set of variables [26,27]. In this study, LT-FA and MGM-FA compositions (dependent variables) were hypothesized to be influenced by diet traits (bromatological and phenolic composition, independent variables). To avoid redundancies, Pearson correlation coefficients were computed separately within independent and dependent variables. When two variables showed Pearson’s correlation above 0.8, only one was retained for subsequent analysis. This pruning step reduced the number of variables from 27 to 9, minimizing collinearity and improving model stability and interpretability. Starting from the original variables (with n and *p*, the number of the two analyzed groups of variables, i.e., LT-FA and bromatological and phenolic diet composition, and n < *p*), CCA derives *n* linear combinations of the original variables, known as canonical variables, from each group (Vn and Wn). The correlation, Cn = Corr (Vn, Wn), between canonical variables is called canonical correlation. The procedure evaluates the relative contribution of each original variable to the derived canonical variable in order to explain the nature of the relationship(s).

The first pair of canonical variables, one for each group, exhibits the largest canonical correlation of any linear correlation of the original variables; the second pair has the largest correlation of the subsequent linear combination and so forth. The obtained Cn were tested for statistical significance using the Wilks’ Lambda test [28], and only the significant ones were considered. Canonical loadings for each significant Cn were then evaluated. These loadings represent the correlations between each original variable and the two canonical variables (Vn and Wn). The canonical correlation maximizes the correlation between two canonical variables (Vn, Wn) which are linear combinations of the original variables. Consequently, a high canonical correlation does not necessarily imply a strong relationship between the two sets of variables. Therefore, when a Cn was significantly different from zero, the redundancy index was calculated to determine how much of the variation in the dependent variables (LT-FA and MGM-FA) was explained by the independent variables (diet data) [29].

Because the animals were group-fed and individual feed intake was not measured, within-animal variability (e.g., feed sorting or selective intake) may have attenuated the observed effects. Therefore, our findings should be interpreted as conservative, highlighting the need for future studies with individual intake monitoring to better characterize intake-driven heterogeneity.

To predict the FA composition of intramuscular fat in LT and MGM from P-FA, partial least square regression (PLSR) was employed due to its capability to handle multi-variate regression models with high collinearity among predictors and to provide more efficient prediction compared to ordinary multivariate regression or principal component regression [30]. PLSR predicts a response matrix Y(n × p) from a predictor matrix X(n × m). In the present study, n represents the number of animals, m the number of fatty acids in plasma, and p the number of fatty acids to be imputed to intramuscular fat of LT and MGM. PLSR extracts a set of orthogonal new variables known as latent factors, which are linear combinations of the original explanatory variables X (fatty acids composition of plasma) that best model the dependent variable Y (fatty acids composition of intramuscular fat in LT and MGM) [31]. To avoid overfitting [30], the maximum number of latent factors was determined with a cross-validation procedure (k-fold). The appropriate number of latent factors was fixed as the one minimizing the root mean square error of prediction in cross-validation (RMSEP-CV) and maximizing the predictive squared correlation coefficient (Q^2^). A more detailed description of the PLSR method can be found in Dimauro et al. [30]. The PLSR analysis was carried out with the pls procedure of R software version 4.1.2 [32]. Finally, the precision and accuracy of the model were assessed, regressing the predicted values against the observed ones. Model precision was evaluated with the coefficient of determination (R^2^), while accuracy was assessed using the Dent and Blackie test [33], which simultaneously evaluates whether the slope of the regression of predicted values differs from 1 and whether the intercept differs from zero, thus indicating whether predictions are unbiased.

## 3. Results

### 3.1. Diet Characteristics

A description of the diet characteristics is reported in Table 2. The breakdown of the herbage on offer into its main botanical components (Table 2) showed that the Gramineae species consistently accounted for two-thirds of the available biomass. The average contribution of the Leguminosae species did not exceed 14.7%, although variable absolute ranges were recorded among farms. The contribution of other dicotyledons ranged, on average, from approximately 18 to 28%.

The bromatological composition of the herbage on offer (Table 2) shows a range of variation among farms for all parameters. The energy level values were in line with advanced and/or reproductive growth stages. The crude protein (CP) content of hay was about half the mean value of herbage, whereas the hay neutral detergent fiber (NDF) content was about 17% higher than herbage (Table 2).

The contents of the phenolic compounds are shown in Table 3. Due to the different contribution of Gramineae, Leguminosae, and other dicotyledons to the whole herbage sample, the content of TotP varied among farms in the pasture-based diets. Herbage on offer showed variability in the TotP, NTP, TP, and ABTS values across farms. Their levels were influenced by both the botanical composition and plant phenological stage. It is worth noting that the hay + concentrate-based diet exhibited TotP contents (Table 3) that were three- to five-fold lower in concentrate and hay, respectively, than the mean value of herbage.

### 3.2. Plasma and Meat Fatty Acids

Table 4 shows the fatty acid composition of plasma and the intramuscular fat of LT (LT-FA) and MGM (MGM-FA). In particular, fatty acids considered important for human health were evaluated. Plasma was characterized by a higher content of ALA, LA, and long-chain derived fatty acids (n-3 FA and n-6 FA) compared to the intramuscular fat of LT and MGM. Conversely, both muscles had a higher content of C18:1n-9 (oleic acid, OA), C18:1 11t (vaccenic acid, VA), cis-9 trans-11-18:2 (CLA), and MUFA than plasma.

### 3.3. Relationships Between Diet Profile and Meat Fatty Acids

Table 5 reports the parameters of the CCA between diet characteristics and fatty acids in LT and MGM. For LT-FA, only the first canonical correlation was statistically significant (C1 = 0.99, *p* = 0.0002), indicating a very strong linear relationship between the two sets of variables. This suggests that the correlation between diet and FA in the LT muscle can be explained by a single pair of canonical variates, V1 and W1. The Wilks’s Lambda value was significantly close to zero, further confirming the strong relationship. Overall, the first canonical correlation was highly significant and explained most of the shared variance between the two variable sets. The results of the CCA can be expressed by the following equations, using standardized coefficients:

V1 = 0.32 EE + 0.46 CP + 0.96 ABTS

and

W1 = 2.38 ALA + 0.66 C18:1 + 0.15 VA + 0.31 CLA + 0.60 SFA − 1.03 n-3/n-6

The absolute values of the standardized canonical coefficients of V1 and W1 suggest that variable ABTS (0.96) for V1, and ALA (2.38), n-3/n-6 PUFA (−1.03), C18:1 (0.66), and SFA (0.6) for W1 are important in forming the first canonical variables. Regarding the correlations between the canonical and the original variables, we can observe that ABTS and EE seemed to be dominant compared to CP, with canonical loadings of 0.78 and 0.51, respectively. Among the LT-FA variables, the ALA, SFA, n-3/n-6 PUFA, and C18:1 are strongly associated with the W1 variable, with correlations of 0.95, −0.7, 0.62, and −0.52, respectively.

For MGM-FA, only the first canonical correlation is statistically significant (C1 = 0.99, *p* = 0.035). The Wilks’s Lambda value close to zero confirmed a strong relationship between MGM-FA and diet quality. The first canonical variable for the feed data group is a weighed sum of

V1 = 0.77 EE + 0.35 CP + 0.43 ABTS

while, for the MGM-FA, it is

W1 = −0.05 ALA − 2.08 C18:1 + 0.50 VA − 0.07 CLA − 0.62 SFA − 0.97 n-3/n-6

The absolute values of the standardized canonical coefficients of V1 and W1 suggest that variable EE (0.77) for V1, and C18:1 (2.08), n-3/n-6 PUFA (0.97), SFA (0.62), and VA (0.5) for W1 are important in forming the first canonical variables. Regarding the correlations between the canonical and the original variables, we can observe that EE seems to be dominant compared to CP and ABTS, with canonical loadings of 0.90, 0.49, and 0.29, respectively. Among the MGM-FA variables, the variable C18:1 is strongly associated with the W1 variable, with a correlation of −0.69, followed by SFA (0.46).

The canonical redundancy index indicated that the first pair of canonical variables accounted for 38% of the variance in LT-FA and 20% in MGM-FA.

### 3.4. Prediction of Meat Fatty Acids from Plasma

The PLSR model was used to predict both LT-FA and MGM-FA from the P-FA composition, extracting five and six latent factors, respectively, in order to minimize the RMSEP in cross-validation (RMSEP-CV). The explained variances by the extracted latent factors are reported in Table S1 (LT-FA) and Table S2 (MGM-FA).

The predicted values were regressed against the observed ones to assess the precision and accuracy of the models. The coefficient of determination (R^2^, precision of models), Dent and Blackie test (*p*-value, accuracy of models), RMSEP in cross-validation (RMSEP-CV), and predictive squared correlation coefficient (Q^2^) are shown in Table 6.

PLSR provided accurate estimates of the predicted values for all dependent variables (Table 6), both for MGM-FA and LT-FA, as confirmed by the Dent and Blackie test (*p*-value > 0.05). The degree of precision of the models (R^2^) of intramuscular MGM-FA ranged from high (R^2^ > 0.8), for almost all fatty acids, to moderate (0.50 < Adj R^2^ < 0.8), for ALA. For the LT muscle, the precision was high for almost all fatty acids and moderate only for MUFAs.

## 4. Discussion

### 4.1. Diet Characteristics

As expected for the sampling period (late spring), herbage on offer showed low CP content values together with high fiber components (NDF, ADF, and ADL) and low IVDMD, in line with the well-documented values in Mediterranean pastures. Variations in phenolic compound contents among farms in the pasture-based diets underlined the high biodiversity of the pasture species composition [16], even over a relatively short distance, and low difference in altitude between the three sites investigated. The ABTS assay, based on the neutralization of stable-colored radicals and widely used to evaluate the antioxidant capacity of compounds, is a common method for assessing the radical scavenging ability of natural products [34,35,36], even in complex biological mixtures such as extracts from the herbage on offer on pastures. Moreover, several studies evidenced that the antioxidant capacity measured by ABTS in various forage plant species is significantly linked to their phenolic content [9,19,37]. Previous studies demonstrated that multispecies swards improve the growth rate of co-grazed cattle and sheep, and, at the same time, grazing multispecies swards can provide multiple benefits for the productivity and environmental sustainability of ruminant production systems [38]. In our trial, we did not directly assess the sward biodiversity; however, it is likely that the farms reflected a degree of multispecies composition typical of natural pastures in the study area [39], which may have contributed to the wide range of outcomes observed across them in herbage on offer (e.g., ABTS values). It has been reported that pasture feeding can enhance the nutritional profile of meat, with higher concentrations of health-promoting phytochemicals in the beef from pasture-fed animals compared to that from concentrate-fed animals [40]. The same authors suggest that forage phytochemicals (antioxidants) could be potential biomarkers in meat products. Our results also suggest that the variation in phenolics and ABTS could be exploited as indicators (e.g., monitoring the feed antioxidant capacity to predict the nutritional traits of animal products) for characterizing different pastures and/or botanical components of herbage, thus adding value to forage resources from natural Mediterranean pastures, and also various fodder sources, as microalgae supplementation [41,42].

### 4.2. Plasma and Meat Fatty Acids

Table 4 highlights the differences between the plasma (P-FA) and muscle (LT-FA and MGM-FA) fatty acids composition. α-linolenic acid (C18:3n-3, ALA) is the precursor of eicosapentanoic acid (C20:5n-3, EPA), docosapentanoic acid (C22:5n-3, DPA), and docosahexaenoic acid (C22:6n-3, DHA), which are synthesized through elongation–desaturation pathways [43]. These long-chain omega 3 fatty acids (EPA, DPA, and DHA) are recognized for their role in preventing atherosclerosis, cardiovascular diseases, and cancer, as well as for supporting brain function and reducing the effects of rheumatoid arthritis [6]. Green pastures are a good source of ALA, as forages such as grasses and clovers contain 50–75% of their total fatty acids as ALA [6,44], as a result of the plants’ unique ability to synthesize this fatty acid de novo. The presence of ALA in animal tissues and plasma originates directly from the diet, and its level depends both on dietary intake and on the ruminal bypass of this fatty acid in an intact form [45].

Linoleic acid (LA, cis-9, cis-12-octadecadienoic acid, C18:2n-6), a precursor of n-6 fatty acids, is involved in the production of eicosanoids (i.e., thromboxanes and leukotrienes), which are related to pro-inflammatory responses [46,47].

As with ALA, the LA content in ruminants depends on the dietary intake and on the fraction that bypasses rumen. Plasma showed higher levels of ALA, LA, and their derived long-chain fatty acids (n-3 FA and n-6 FA) compared with LT and MGM muscles. This reflects the differences in the fate and metabolic pathway, depending on whether they are incorporated into plasma or tissues.

CLA is a collective term for a group of positional and geometric isomers of linoleic acid. The most abundant isomer, cis-9, trans-11 CLA [48], is an intermediate in the ruminal bio-hydrogenation of linoleic acid and has vaccenic acid (C18:1 11t, VA) as its precursor.

The bio-hydrogenation of dietary ALA and LA in the rumen also produces vaccenic acid (C18:1 11t, VA), which is a major precursor of CLA via desaturation catalyzed by the Δ9-desaturase enzyme [49]. This mechanism is responsible for the higher content of C18:1 n-9 (Oleic Acid, OA) and CLA in LT and MGM muscles compared to plasma, since both fatty acids are produced in tissues by Δ9-desaturase from stearic acid (C18:0) and VA, respectively. These differences were partially expected, since it is known that the de novo synthesis of fatty acids also occurs at the tissue level (e.g., CLA synthesized by the animal tissues from VA, of endogenous origin [50]), causing the muscle fatty acid composition to diverge somewhat from that of plasma.

LT and MGM tissue differed in LA, VA, UFA, and n-6 FA; MGM had a higher LA and n-6 FA, while LT had a higher VA and UFA. According to Wood et al. [51], the differences between muscles in fatty acid composition may reflect the differences in muscle fiber type, because “red” muscles (MGM) are richer in phospholipids than “white” muscles (LT).

The n-3/n-6 PUFA ratio is widely used as an index to evaluate the nutritional value of fat for human consumption, since a low n-3/n-6 ratio is considered as a major risk factor for the development of cardiovascular diseases, cancer, and inflammatory and autoimmune disorders [52].

### 4.3. Relationships Between Diet Profile and Meat FA Variables

In our study, CCA was used to investigate the multivariate relationships between the diet composition and fatty acid profile of two muscles, *Longissimus thoracis* (LT-FA) and *Musculus gluteus maximus* (MGM-FA), in animals subjected to different feeding regimens. This approach allowed the identification of linear combinations of variables from each set that are maximally correlated.

As reported in previous studies [53,54], only standardized canonical coefficients exceeding an absolute value of 0.5 were considered relevant for interpretation. In the LT muscle, ABTS and EE emerged as the most influential dietary components, while ALA, the n-3/n-6 PUFA ratio, C18:1, and SFA were the key contributors among fatty acids traits. This suggests that the antioxidant capacity (as reflected by ABTS) and ether extract (EE) may modulate the deposition or metabolism of specific fatty acid classes, particularly those involved in inflammatory and metabolic processes.

These findings align with studies indicating that the dietary antioxidants and lipid content can significantly affect the intramuscular fat composition [49,51]. In particular, ALA is often used as a dietary marker of pasture-based feeding systems, and its high canonical loading (0.95) underscores its central role in differentiating lipid profiles among diets.

The inverse relationship observed between SFA and ALA (loading −0.69 vs. 0.95) may reflect the well-documented shift from saturated to unsaturated fatty acids in animals fed forage-based diets rich in n-3 precursors [3,44]. The positive loading of the n-3/n-6 PUFA ratio further supports this interpretation and suggests a more favorable nutritional profile, with potential benefits for human health [55].

Although canonical loadings must be interpreted cautiously, they remain useful for identifying the original variables most associated with each canonical function. In our analysis, the canonical variable W1 for the LT muscle clearly represents a pattern characterized by a higher ALA and n-3 PUFA and a lower SFA, which could be indicative of an improved lipid quality in the meat.

The canonical redundancy index of 38% confirmed that a substantial proportion of the variance in the fatty acid profile was explained by dietary variables, exceeding the commonly accepted 20% threshold for meaningful interpretation [56]. This reinforces the robustness of the association and the importance of diet composition in shaping meat lipid characteristics.

Similarly to Kearney et al. [40], our findings demonstrate that ABTS, which correlates with the phenolic content, serves as a reliable indicator of meat quality. This not only confirms the established knowledge that increasing ABTS levels (and, thus, phenolics) are associated with LT-FA profiles richer in ALA and n-3/n-6 ratios and lower in SFA and C18:1, but also provides indirect validation of the proposed approach, i.e., using CCA as a preliminary tool to predict the intramuscular fatty acid composition in beef based on proximate feed analyses.

In MGM muscle, EE, C18:1, the n-3/n-6 PUFA ratio, SFA, and VA were the most important variables forming the first canonical variable. EE was strongly correlated with V1, while C18:1 and SFA were most strongly associated with W1. The redundancy index of MGM (0.20) was lower than that of LT (0.38), indicating a weaker relationship between the two variable sets in this muscle.

Overall, our results suggest that animals consuming diets with a higher antioxidant capacity (captured by ABTS) and rich in n-3 precursors (EE fraction) tended to deposit healthier fat profiles in muscle, with higher levels of ALA and a more favorable n-3/n-6 ratio, while reducing SFA. This pattern is consistent with pasture-based feeding and highlights the potential nutritional advantages of diets supplying both antioxidant activity and unsaturated fatty acid precursors.

While the results related to LT-FA are consistent with the existing literature [49,51], the mechanism underlying MGM-FA requires further investigation. Nevertheless, the fatty acid composition of the analyzed muscles (Table 4), rich in polyunsaturated fatty acids, reflects the grazing-derived characteristics of the diet. In this context, the positive relationship between dietary EE and the n-3/n-6 ratio observed in both muscles can be explained by the high content of n-3 precursors in EE. This may also account for the inverse relationship between EE and C18:1, since this fatty acid tends to increase in grain-based diets and decrease in grass-based diets [57]. The relationship between EE and SFA appeared to differ between the two muscles. In this case, the differences in muscle fiber type may play a role.

However, the validity of the proposed CCA analysis is confirmed and also represents a methodological novelty. Unlike traditional univariate or bivariate analyses, which test one relationship at a time, CCA simultaneously considers sets of diet and meat variables, identifying integrated patterns rather than isolated associations. This multivariate perspective strengthens its role as an exploratory predictive tool, broadening the understanding of how dietary traits shape beef lipid profiles. It can also guide further research that sheds new light on the mechanisms behind the formation of the acid composition of bovine intramuscular fat.

On the whole, the results suggest a significant and meaningful relationship between the two sets of variables, diet characteristics, and meat fatty acid, with certain variables contributing more to this relationship than others according to the muscle investigated.

Investigating the relationship between the diet composition and meat fatty acid profile is helpful for understanding the dynamic metabolic processes that regulate the lipid assimilation, transport, and deposition in muscle tissue of beef cattle. Strong correlations confirm that the dietary nutrients and antioxidant capacity directly affect lipid metabolism before muscle deposition. The link between the diet composition, circulating fatty acids, and muscle composition reinforces the importance of diet characteristics in modulating meat lipid profiles which are relevant for both animal nutrition strategies and meat quality optimization.

### 4.4. Prediction of Meat Fatty Acids from Plasma

PLSR was applied to predict fatty acids in both MGM and LT muscles from fatty acids analyzed in plasma. These results are consistent with other studies [15] that use simple linear regression models to predict tissues’ fatty acid profiles, such as the n3/n6 PUFA ratio. The added value of the PLSR model is that it has a high accuracy for a wider range of fatty acids than previously reported, although the precision varied according to the fatty acid or muscle involved. Fatty acids contribute significantly to various aspects of meat quality and nutritional value, but their evaluation usually requires the slaughter of the animal. Since drawing blood from cattle is a relatively simple and minimally invasive practice, predicting the meat FA composition based on plasma represents a valuable advancement. Despite the limited number of animals used in the analysis, the PLSR procedure provided estimates with a good degree of precision and accuracy for many relevant fatty acids in two different muscles. Therefore, this model represents a promising tool to predict the meat fatty acid composition of cattle from blood. It should be noted, however, that no independent external validation set could be used due to the limited number of animals available. Although cross-validation provided robust estimates of the predictive ability, this remains a limitation, and further work with larger and more diverse populations is needed to confirm the generalizability of the models.

## 5. Conclusions

This study demonstrates that plasma fatty acids and dietary traits can serve as predictors of intramuscular fatty acid profiles in beef cattle. Canonical correlation analysis emerged as a valuable exploratory tool, highlighting the integrated relationships between dietary characteristics (lipid fraction and antioxidant capacity) and intramuscular fatty acid profiles in the *Longissimus dorsi* and *Musculus gluteus maximus* of young beef cattle finished under stall and pasture systems.

In parallel, Partial Least Squares Regression confirmed the predictive potential of plasma markers for specific fatty acids of nutritional interest (e.g., α-linolenic acid, docosapentaenoic acid, and the n-3/n-6 ratio), managing multicollinearity among variables. The results suggest that plasma fatty acid profiling represents a promising non-invasive proxy for estimating the intramuscular fatty acid composition of beef, although its practical application requires further validation, particularly concerning the time of blood sampling relative to slaughter.

The association observed between dietary bioactive compounds with antioxidant properties (e.g., ABTS assay), and favorable fatty acid profiles in beef supports the hypothesis that feeding strategies could contribute to improve beef nutritional quality.

These findings provide a proof of concept for the combined use of CCA and PLSR as complementary multivariate approaches to link diet, plasma, and meat fatty acid composition. Future research should include larger and more diverse cattle populations, multiple production systems, and repeated measurements over time to confirm these insights and assess their reproducibility and practical value for on-farm decision making.

## Figures and Tables

**Table 2:** Herbage availability, botanical composition, and feed quality in pasture and hay + concentrate diets.

		Pasture Diet			Hay + Concentrate Diet	
	Unit	Farm 1	Farm 2	Farm 3	Hay	Concentrate
Herbage on offer	t ha^−1^ DM	1.1 ± 0.18	1.4 ± 0.15	2.1 ± 0.16	-	-
Gramineae	% FM	82.1 ± 6.83	66.8 ± 5.68	65.5 ± 5.91	-	-
Leguminosae	% FM	0.3 ± 3.11	4.4 ± 2.59	14.7 ± 2.70	-	-
Other dicotyledons	% FM	17.7 ± 6.02	28.7 ± 5.01	20.2 ± 5.22	-	-
CP	% DM	7.6 ± 0.50	10.4 ± 0.42	9.5 ± 0.44	5.03 ± 0.18	16.52 ± 1.15
NDF	% DM	66.5 ± 1.66	55.5 ± 1.38	58.5 ± 1.44	70.13 ± 0.88	18.75 ± 0.81
ADF	% DM	38.1 ± 0.86	32.6 ± 0.72	34.8 ± 0.74	41.5 ± 0.61	10.76 ± 0.09
ADL	% DM	3.5 ± 0.37	3.7 ± 0.31	3.8 ± 0.32	3.24 ± 0.09	1.16 ± 0.12
EE	% DM	2.3 ± 0.09	2.5 ± 0.08	1.9 ± 0.08	1.42 ± 0.08	2.95 ± 0.52
IVDMD	% DM	46.3 ± 2.24	55.4 ± 1.86	55.4 ± 1.94	50.25 ± 0.75	96.53 ± 0.54
Energy level	MJME/kg DM	7.6 ± 0.15	8.1 ± 0.12	7.8 ± 0.13	6.95 ± 0.81	12.89 ± 0.62

All values are expressed as lsmeans ± SE. Energy levels of diets estimated using the Large Ruminant Nutrition System software, version 1.0.32, http://nutritionmodels.com/lrns.html. Abbreviations: CP = crude protein, NDF = neutral detergent fiber, ADF = acid detergent fiber, ADL = acid detergent lignin, EE = ether extract, and IDMD = in vitro dry matter digestibility.

**Table 3:** Total phenolics (TotP), non-tannic phenolics (NTP), tannic phenolics (TP), and total antioxidant capacity (TEAC) by ABTS method in pasture components and hay + concentrate diets across farms.

		TotP (g GAE kg^−1^ DW)	NTP (g GAE kg^−1^ DW)	TP (g GAE kg^−1^ DW)	ABTS (mmol TEAC 100 g^−1^ DW)
Herbage on offer	Farm 1	9.6 ± 0.7	5.1 ± 0.4	4.5 ± 0.5	4.9 ± 0.3
	Farm 2	17.3 ± 1.4	9.1 ± 0.9	8.3 ± 0.6	9.1 ± 0.6
	Farm 3	13.5 ± 0.6	7.5 ± 0.5	6.0 ± 0.5	7.2 ± 0.3
Gramineae	Farm 1	6.7 ± 0.2	4.0 ± 0.3	2.7 ± 0.4	3.6 ± 0.01
	Farm 2	13.7 ± 0.7	7.7 ± 0.5	6.0 ± 0.5	6.7 ± 0.1
	Farm 3	7.2 ± 0.1	4.4 ± 0.4	2.8 ± 0.3	3.6 ± 0.1
Leguminosae	Farm 1	26.3 ± 1.7	6.9 ± 0.5	19.4 ± 1.2	13.6 ± 0.4
	Farm 2	20.0 ± 0.6	9.6 ± 0.4	10.3 ± 0.5	10.3 ± 0.6
	Farm 3	17.1 ± 0.2	9.1 ± 0.4	8.0 ± 0.1	7.1 ± 0.4
Other dicotyledons	Farm 1	23.1 ± 0.7	10.0 ± 0.5	13.1 ± 0.6	10.7 ± 0.3
	Farm 2	25.6 ± 0.2	11.8 ± 0.8	13.7 ± 0.6	14.4 ± 0.2
	Farm 3	31.2 ± 0.6	16.2 ± 0.3	15.0 ± 0.7	18.9 ± 0.5
Hay +	Stall	4.3 ± 0.1	3.6 ± 0.2	0.7 ± 0.1	1.6 ± 0.1
Concentrate	Stall	3.0 ± 0.01	1.9 ± 0.03	1.0 ± 0.03	1.4 ± 0.05

All values are expressed as means ± SE.; GAE: gallic acid equivalent; TEAC: Trolox equivalent antioxidant capacity.

**Table 4:** Fatty acid profile (mol%) in plasma and muscles of F1 Limousine × Sardo-Bruna cattle.

Fatty Acid	LT-FA *	MGM-FA *	P-FA *	S.E.	*p* Value
C18:3n-3, ALA	1.22 a (0.21–2.86)	1.45 a (0.11–3.73)	4.87 b (0.48–9.94)	0.66	<0.001
C18:2n-6, LA	5.54 a (2.37–11.99)	10.05 b (4.00–18.43)	25.77 c (15.76–40.15)	1.46	<0.001
C18:1n-9, OA	31.7 a (23.56–37.54)	27.6 a (14.13–40.11)	7.5 b (5.24–11.08)	1.87	<0.001
C18:1 11t, VA	4.01 a (2.26–6.33)	2.78 b (1.48–4.76)	0.88 c (0.51–1.53)	0.29	<0.001
C22:5n-3, DPA	0.50 (0.05–1.55)	0.85 (0.11–2.29)	0.84 (0.34–1.30)	0.17	0.26
CLA cis-9 trans-11-18:2	1.25 a (0.51–1.80)	1.10 a (0.65–1.58)	0.36 b (0.13–0.73)	0.09	<0.001
UFAs	50.5 a (44.50–62.34)	53.1 b (45.93–61.98)	48.2 ab (32.94–56.16)	1.6	0.02
SFAs	49.5 (37.66–55.50)	46.9 (38.02–54.07)	51.7 (43.79–66.79)	1.6	0.13
MUFAs	40.1 a (31.48–46.24)	34.5 a (18.03–49.68)	10.2 b (6.90–13.15)	2.2	<0.001
n-3/n-6 PUFA	0.22 (0.08–0.33)	0.18 (0.08–0.30)	0.25 (0.03–0.42)	0.03	0.41
n-3 FA	1.86 b (0.28–5.00)	2.80 b (0.71–6.42)	6.94 a (1.35–13.19)	0.89	<0.001
n-6 FA	7.21 c (3.04–15.81)	13.93 b (5.71–24.50)	30.84 a (20.21–45.03)	1.69	<0.001

* LT = fatty acid composition of intramuscular fat in *Longissimus thoracis*; MGM = fatty acid composition of intramuscular fat in *Musculus gluteus maximus*. Different letters within rows indicate significant differences (*p* < 0.05); absence of letters denotes non-significant differences. Abbreviations: ALA = α-linolenic acid; LA = linoleic acid; OA = oleic acid; VA = vaccenic acid; DPA = docosapentaenoic acid; CLA = conjugated linoleic acid; UFAs = unsaturated fatty acids; SFAs = saturated fatty acids; MUFAs = monousaturated fatty acids.

**Table 5:** Canonical correlation parameters between diet traits and fatty acids in LT and MGM muscles.

	LT		MGM	
	Standardized Canonical Coefficient	Canonical Loading	Standardized Canonical Coefficient	Canonical Loading
Diet Characteristics	V1		V1	
EE	0.3181	**0.5084**	**0.7677**	**0.9139**
CP	0.4567	0.1973	0.3471	0.4967
ABTS	**0.9645**	**0.7757**	0.4354	0.2893
Fatty acids	W1		W1	
C18:3n-3, ALA	**2.3766**	**0.9482**	−0.0475	0.3484
C18:1, Oleic acid	**0.6618**	**−0.5218**	**−2.0763**	**−0.6907**
C18:1 11t, VA	0.1520	0.3023	**0.5017**	0.0143
cis-9 trans-11, CLA	0.3144	0.3720	−0.0712	**−0.6176**
Saturated fatty acids, SFAs	**0.6031**	**−0.6992**	**−0.6220**	0.4635
n-3/n-6 PUFA ratio	**−1.0354**	**0.6271**	**−0.9734**	0.1853
Canonical correlation (C1)	0.99		0.99	
Wilks’s Lambda	0		0	
P	0.0002		0.035	

V1 = canonical variable for diet characteristics; W1 = canonical variable for fatty acids in LT and MGM. Standardized canonical coefficients > 0.50 (in bold) highlight the predictors with the greatest influence in the canonical model. Canonical loadings represent the correlation of each original variable with the canonical function. Loadings > 0.50 (in bold) indicate the variables most strongly associated with that function.

**Table 6:** Precision, accuracy, and root mean square error of prediction (RMSEP) and predictive squared correlation coefficient (Q^2^) of PLSR models predicting LT-FA and MGM-FA from plasma fatty acids.

Fatty Acids	LT-FA				MGM-FA			
	Dent and Blackie Test (*p*-Value)	Coefficient of Determination (R^2^)	RMSEP-CV	Q^2^	Dent and Blackie Test (*p*-Value)	Coefficient of Determination (R^2^)	RMSEP-CV	Q^2^
C18:3n-3, ALA	ns	0.94	0.24	0.94	ns	0.64	0.77	0.70
C18:2, LA	ns	0.87	1.21	0.88	ns	0.95	0.16	0.99
C18:1	ns	0.82	1.47	0.89	ns	0.98	1.03	0.99
C18:1 11t, VA	ns	0.81	0.44	0.88	ns	0.97	0.15	0.97
C22:5n-3, DPA	ns	0.86	0.20	0.87	ns	0.97	0.10	0.98
CLA cis-9 trans-11	ns	0.90	0.11	0.91	ns	0.91	0.06	0.93
Saturated Fatty Acids (SFAs)	ns	0.83	1.99	0.85	ns	0.96	0.90	0.96
n-3/n-6 ratio	ns	0.96	0.02	0.96	ns	0.92	0.02	0.92
n-3-series fatty acids	ns	0.92	0.49	0.92	ns	0.97	0.37	0.97
n-6-series fatty acids	ns	0.88	1.55	0.88	ns	0.94	0.25	0.99
Mono Unsaturated Fatty Acids (MUFAs)	ns	0.77	1.66	0.86	ns	0.97	1.25	0.99

ns = not significant.

## Data Availability

The data are available upon request.

## Supplementary Materials

The following supporting information can be downloaded at: https://www.mdpi.com/article/10.3390/ani15202969/s1, Table S1: Explained variance of LT fatty acids by latent factors in multivariate analysis; Table S2: Explained variance of MGM fatty acids by latent factors in multivariate analysis.

## Abbreviations

The following abbreviations are used in this manuscript:

PUFA	n-3 polyunsaturated fatty acid
LW	live weight
DM	dry matter
CP	crude protein
NDF	neutral detergent fiber
ADF	acid detergent fiber
ADL	acid detergent lignin
EE	ether extract
IVDMD	in vitro dry matter digestibility
ToP	total phenolics
NTP	non-tannic phenolics
TP	tannic phenolics
GAE	gallic acid equivalent
ABTS	2,2′azinobis (3-ethylbenzothiazoline-6-sulphonic acid) diammonium salt
TEAC	Trolox equivalent antioxidant capacity
LT	*Longissimus thoracis*
MGM	*Musculus gluteus maximus*
P-FA	plasma fatty acid composition
CCA	canonical correlation analysis
LT-FA	*Longissimus thoracis* fatty acid composition
MGM-FA	*Musculus gluteus maximus* fatty acid composition
Vn	canonical variable for diet characteristics
Wn	canonical variable for fatty acids in LT and MGM
PLSR	Partial Least Square Regression
RMSEP	root mean square error of prediction
MES	Model Evaluation System
ALA	α-linolenic acid
LA	linoleic acid
OA	oleic acid
VA	vaccenic acid
DPA	docosapentaenoic acid
CLA	conjugated linoleic acid
UFAs	unsaturated fatty acids
SFAs	saturated fatty acids
MUFAs	monounsaturated fatty acids
n-3 FA	omega-3 fatty acid
n-6 FA	omega-6 fatty acid
EPA	eicosapentanoic acid (C20:5n-3)
DPA	docosapentanoic acid (C22:5n-3)
DHA	docosahexaenoic acid (C22:6n-3)
