# Supplementary material for: Predicting Beef Fatty Acid Composition from Diet and Plasma Profiles Using Multivariate Models
Source: Animals (Basel). 2025 Oct 14;15(20):2969. doi: 10.3390/ani15202969 (PMC12560911; doi:10.3390/ani15202969)
Supplement: Supplementary file 1 [file animals-15-02969-s001.zip › animals-3898730-supplementary.pdf]

Supplementary Materials

Predicting Beef Fatty Acid Composition from Diet and Plasma Profiles Using Multivariate Models

Marco Acciaro [1], Leonardo Sulas [2], Gianfranca Carta [3], Sebastiano Banni [3], Elisabetta Murru [3], Claudia Manca [3], Corrado Dimauro [4], Myriam Fiori [1], Andrea Cabiddu [1], Giovanni Antonio Re [2], Maria Giovanna Molinu [5], Giovanna Piluzza [2]* and Valeria Giovanetti [1]

[1] AGRIS Sardegna, S.S. Sassari-Fertilia 291, km 18.6, 07100, Sassari, Italy;
[2] Institute for the Animal Production System in Mediterranean Environment, National Research Council, Traversa La Crucca 3, località Baldinca, 07100, Sassari, Italy
[3] Department of Biomedical Sciences, Section of Physiology, University of Cagliari, Cittadella Universitaria, 09040 Monserrato, Cagliari, Italy;
[4] Università degli studi di Sassari, via E. de Nicola, 07100, Sassari, Italy
[5] Institute of Sciences of Food Production, National Research Council, Traversa La Crucca 3, località Baldinca, 07100, Sassari, Italy
* Correspondence: giovanna.piluzza@cnr.it; Tel.: +39 0792841608

Supplementary Table S1. Explained variance of LT fatty acids by latent factors in multivariate analysis.

	Latent factors				
**LT-FA**	**1**	**2**	**3**	**4**	**5**
C18:3n-3, ALA	70,75	78,38	78,51	94,30	94,32
C18:2n-6, LA	61,56	68,39	78,15	87,03	87,03
C18:1n-9	29,52	56,47	57,06	72,33	81,74
C18:1 11t, VA	9,81	45,98	56,96	77,17	81,35
C22:5n-3, DPA	66,3	77,63	78,00	86,39	86,39
CLA cis-9 trans-11	51,29	59,08	71,5	84,91	89,85
Unsatured Fatty Acids (UFA)	29,38	73,54	73,61	74,93	82,99
Satured fatty Acids (SFA)	29,38	73,54	73,61	74,93	82,99
n-3/n-6 PUFA ratio	69,72	69,82	88,79	96,03	96,49
n-3-PUFA	70,25	79,22	79,22	91,75	91,83
n-6-PUFA	63,56	70,29	79,3	87,62	87,64
Mono Unsatured Fatty Acids (MUFA)	31,54	49,57	55,53	66,21	77,54

Supplementary Table S2. Explained variance of MGM fatty acids by latent factors in multivariate analysis.

	latent factors					
**MGM-FA**	**1**	**2**	**3**	**4**	**5**	**6**
C18:3n-3, ALA	35,49	38,31	46,73	46,77	64,22	64,36
C18:2n-6, LA	62,01	65,07	75,72	80,18	93,52	95,03
C18:1n-9	59,40	67,91	88,87	94,99	97,05	97,92
C18:1 11t, VA	18,57	72,63	72,86	83,94	86,80	96,71
C22:5n-3, DPA	60,68	61,82	81,52	81,60	95,56	96,65
CLA cis-9 trans-11	3,80	44,80	45,59	47,14	87,61	91,18
Unsatured Fatty Acids (UFA)	29,72	64,62	82,63	87,47	90,50	90,51
Satured fatty Acids (SFA)	12,60	57,20	75,48	87,49	91,22	95,62
n-3/n-6 PUFA ratio	54,19	55,95	82,41	89,27	89,52	92,08
n-3-PUFA	61,61	61,65	84,83	85,85	93,37	96,64
n-6-PUFA	63,21	65,99	74,46	78,98	91,15	93,85
Mono Unsatured Fatty Acids (MUFA)	55,92	69,19	89,75	95,04	97,40	97,54
